# A metal artifact reduction algorithm in CT using multiple prior images by recursive active contour segmentation

**DOI:** 10.1371/journal.pone.0179022

**Published:** 2017-06-12

**Authors:** Haewon Nam, Jongduk Baek

**Affiliations:** 1 Department of Liberal Arts and Science, Hongik University, Sejong, South Korea; 2 Yonsei Institute of Convergence Technology, Yonsei University, Incheon, South Korea; 3 School of Integrated Technology, Yonsei University, Incheon, South Korea; Beijing University of Technology, CHINA

## Abstract

We propose a novel metal artifact reduction (MAR) algorithm for CT images that completes a corrupted sinogram along the metal trace region. When metal implants are located inside a field of view, they create a barrier to the transmitted X-ray beam due to the high attenuation of metals, which significantly degrades the image quality. To fill in the metal trace region efficiently, the proposed algorithm uses multiple prior images with residual error compensation in sinogram space. Multiple prior images are generated by applying a recursive active contour (RAC) segmentation algorithm to the pre-corrected image acquired by MAR with linear interpolation, where the number of prior image is controlled by RAC depending on the object complexity. A sinogram basis is then acquired by forward projection of the prior images. The metal trace region of the original sinogram is replaced by the linearly combined sinogram of the prior images. Then, the additional correction in the metal trace region is performed to compensate the residual errors occurred by non-ideal data acquisition condition. The performance of the proposed MAR algorithm is compared with MAR with linear interpolation and the normalized MAR algorithm using simulated and experimental data. The results show that the proposed algorithm outperforms other MAR algorithms, especially when the object is complex with multiple bone objects.

## Introduction

Metal artifacts are one of the most common problems in computed tomography (CT). Various metal implants in the human body such as dental fillings [1], orthopedic implants [2], hip prostheses [3], and implanted marker bins [4], generate dark and bright streaks owing to the high attenuation of metals, which degrade the image quality and diagnostic value of CT images. To reduce metal artifacts, several metal artifact reduction (MAR) algorithms have been developed [5]. These are classified into two groups: projection completion methods and iterative methods.

Projection completion methods treat projections through the metal trace region as missing data and estimate them using neighboring measured data by linear [6], higher-order [7–9], wavelet [10, 11], prior knowledge [12, 13], and diffusion [14, 15] approximations. Interpolation-based MAR algorithms are simple, but produce additional artifacts when large metal implants are present. To prevent this, MAR algorithms utilizing a prior image have been suggested [16–20]. In these algorithms, the metal trace region is replaced by the forward projection of the prior image obtained by applying thresholding on the pre-corrected CT images [16–19] with the assumption that pixel values do not vary substantially within materials. Instead of using simple thresholding, Zhang *et* *al*. [20] used a TV minimization regularized algebraic reconstruction technique (ART) to find the prior image. In general, MAR algorithms with prior images demonstrate better performance in artifact reduction, but acquiring a high quality prior image is essential.

Iterative methods attempt to iteratively reduce the mismatch between measured data and forward projection of the object [21–27]. By excluding data in the metal trace region of the sinogram, MAR with iterative methods becomes an ill-posed problem that is stabilized by using a priori knowledge of the image as a regularization term [21–24]. Wang *et* *al*. [21] proposed two iterative deblurring algorithms that used an expectation maximization (EM) formula and ART adapted for MAR. Williamson *et* *al*. [22] proposed statistical iterative reconstruction using prior information about the geometry of metal objects and the detector response model. Choi *et* *al*. [23] and Zhang *et* *al*. [24] proposed iterative MAR based on constrained optimization with total variation (TV) and a quadratic smoothness function as a regularizer, respectively. Verburg and Seco [25] used a regularized iterative method only applied on a high-Z implants traced sinogram. Lemmens *et* *al*. [26] used maximum a posteriori (MAP) reconstruction with thresholding-based multimodal prior, and Nasirudin et al. [27] used material decomposed images of spectral CT as priors, and reduced the metal artifacts by regularized maximum likelihood approach. Compared to the projection completion methods, MAR with iterative methods are robust to noise because they are controlled by a regularization term. However, the high computational cost is a practical obstacle.

The proposed MAR algorithm is categorized in the projection completion group. To reduce estimation errors in the metal trace region, we generate multiple prior images by applying a recursive active contour (RAC) segmentation technique to the MAR image with linear interpolation [6], where the number of prior image is controlled by the RAC depending on the object complexity. A sinogram basis is acquired by conducting forward projection of prior images. The metal trace region of the original sinogram is then replaced by the linearly combined sinogram of prior images. When the object is heterogeneous and the data acquisition condition is not ideal, residual errors occur between the original sinogram and the linearly combined sinogram. Additional correction is performed to compensate. We iteratively continue the process until the norm of the residual errors is minimized. As a result, the proposed algorithm finds good prior images and effectively reduces metal artifacts. To evaluate the performance of the proposed MAR algorithm, both numerical and physical experiments are conducted. Quantitative evaluations are performed and compared with the MAR algorithm with linear interpolation (LIN) [6] and normalized MAR (NMAR) algorithm [18].

## Materials and methods

In this section, we briefly review the RAC segmentation technique and then describe the proposed MAR algorithm.

### Recursive active contour segmentation

RAC segmentation is based on classical active contour segmentation proposed by Chan-Vese [28]. The active contour model uses a level set function to divide an image into two separate regions.

Let *I* be a given image on $\Omega \subset R^{3}$ such that I:Ω→R. The active contour model minimizes the following equation [28]:

$$\begin{array}{c} \min_{\varphi }F\left(\varphi \right)=\int_{\Omega }\{|I-c_{1}{|}^{2}H\left(\varphi \right)+|I-c_{2}{|}^{2}\left(1-H\left(\varphi \right)\right)\}+\mu \int_{\Omega }DH\left(\varphi \right), \end{array}$$ (1)

where *H* is the Heaviside function and *D* is the distributional derivative. A function *φ* is the levelset function which separates an image into two subregions, and *c*_1_ and *c*_2_ are mean values of corresponding subregions; *μ* is a regularization parameter to control the length of the active contour and segmentation sensitivity.

**Fig 1:**
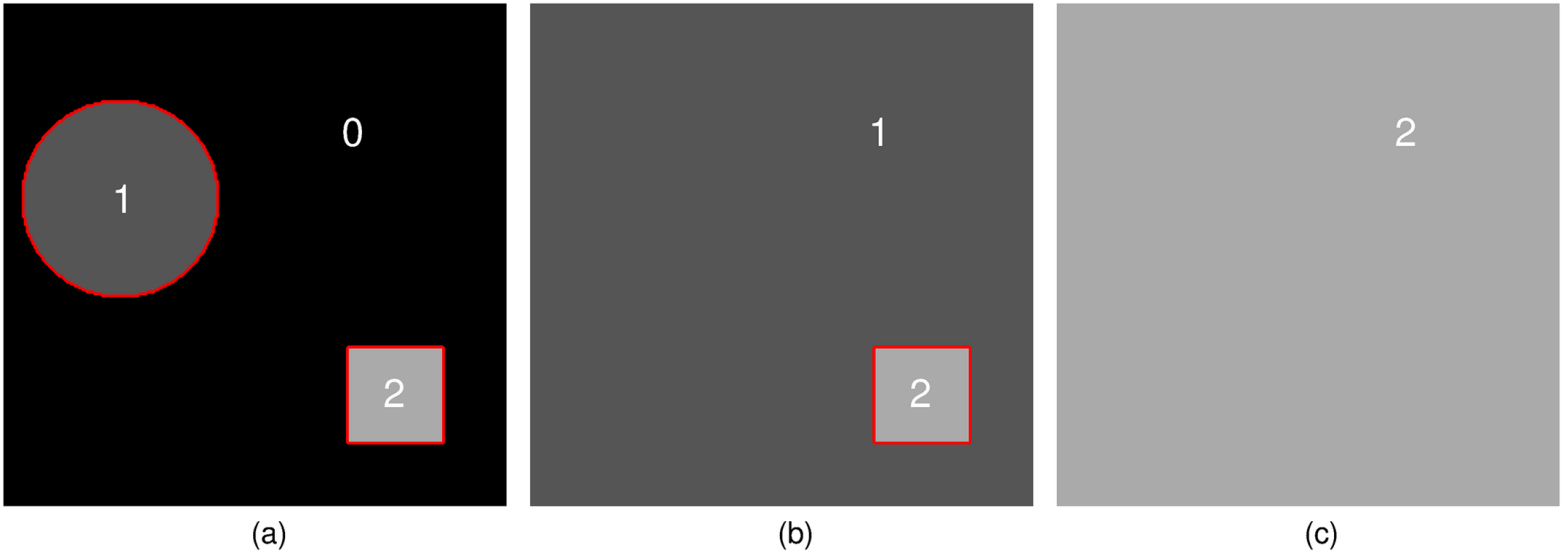
Concept of recursive active contour segmentation. (a) *I*_0_: initial image, (b) *I*_1_: image obtained by replacing the background values of *I*_0_ with one, and (c) *I*_2_: image by replacing the background values of *I*_1_ with two.

Image segmentation into multiple subregions can be performed by recursively applying the active contour model. Suppose image *I*_0_ has two objects with different intensities as shown in Fig 1(a). By applying the active contour model to *I*_0_, two objects are separated together from the background by the levelset function. To further segment subregions, the background values in *I*_0_ are replaced by the minimum value of the foreground regions in *I*_0_. Then a new image *I*_1_ is acquired as shown in Fig 1(b). By continuing the segmentation process until the entire image is filled with a constant as shown in Fig 1(c), multiple subregions are segmented. Note that red lines in Fig 1 indicate a region where the values of the levelset function are zero. The levelset function has positive values in the foreground regions.

**Fig 2:**
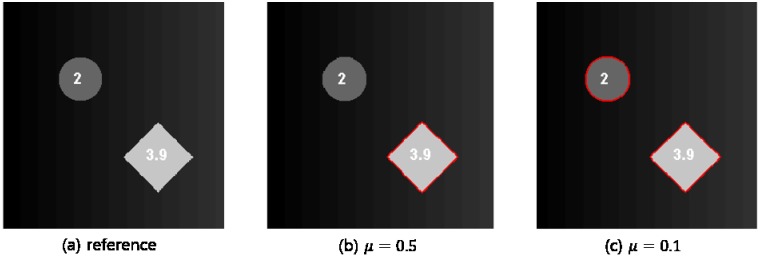
Segmentation results with different regularization parameter *μ*.

The main advantage of the RAC technique is that the number of subregions is determined adaptively during the segmentation process. Although we describe image segmentation using a piecewise linear object, the RAC technique is still applicable to heterogeneous objects by controlling the regularization parameter *μ* in Eq (1) [29]. Since the regularization parameter controls the segmentation sensitivity [29], small *μ* is needed for the segmentation of low contrast intensity object, which increases the number of segmented subregions. Fig 2 illustrates the segmentation results depending on the regularization parameter, where the background intensity increases gradually from zero to one toward the right direction.

### Proposed method

**Fig 3:**
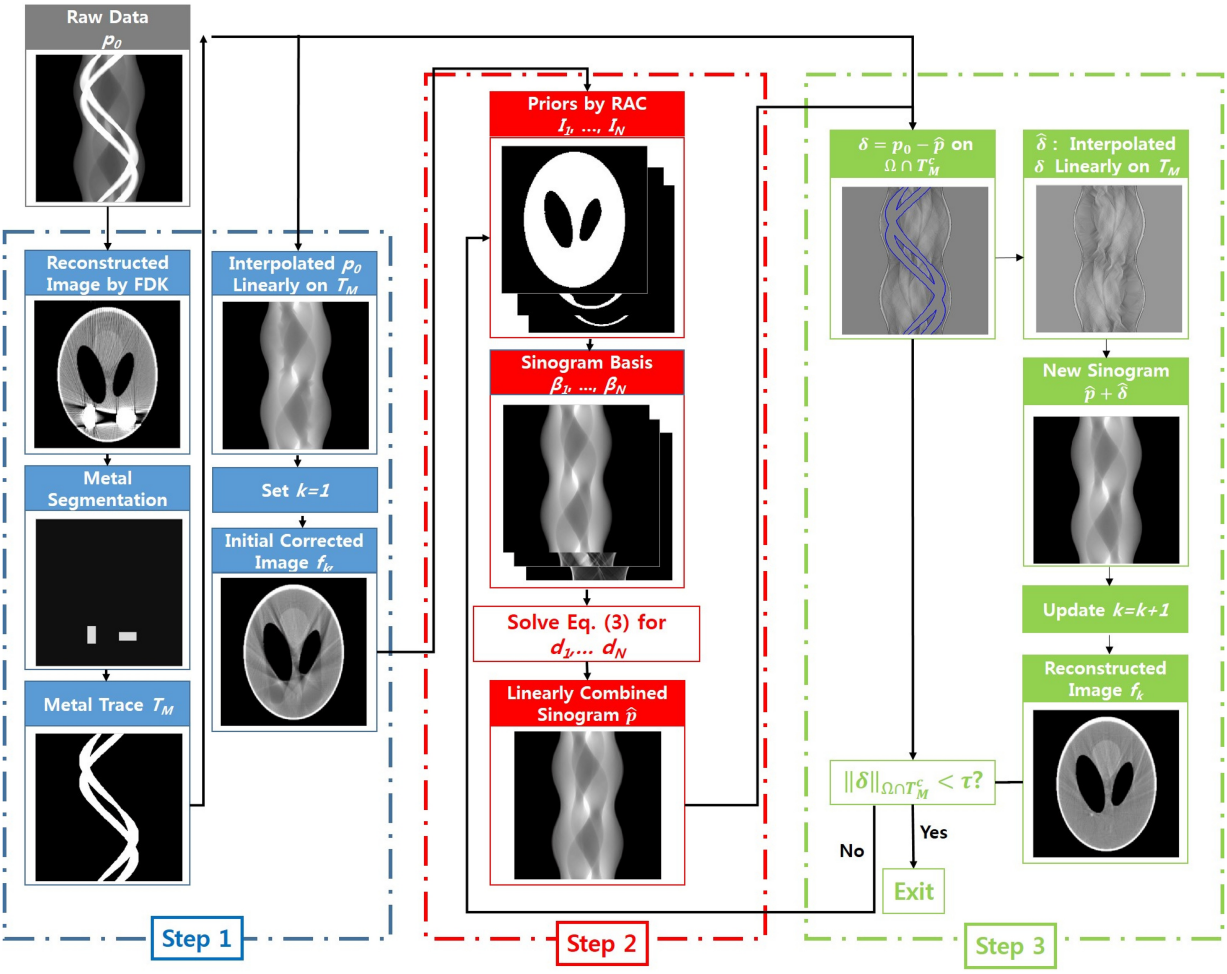
Flowchart of the proposed algorithm.

The proposed MAR algorithm consists of three steps as described in Fig 3. In the first step, the initial image is reconstructed by the Feldkamp, David and Kress (FDK) algorithm using the original sinogram *p*_0_, and metal regions are segmented by the active contour [28]. Note that the active contour technique is more effective in metal segmentation than the simple thresholding technique when multiple metals are present. Then, the metal trace region, *T*_*M*_, is identified by the forward projection of the segmented metals within the sinogram domain Ω. The initial corrected image, *f*_*k*_ for *k* = 1, is acquired by applying linear interpolation on the metal trace region *T*_*M*_ [6].

In the second step, *f*_*k*_ is divided into *N* subregions, *I*_1_, …, *I*_*N*_ by RAC. In our implementation, we chose the regularization parameter in Eq (1) as 0.1, and the number of subregions *N* is determined accordingly. The segmented subregions are used as prior images, which have the intensity as one; thus, finding the proper coefficient *d*_*j*_ for each subregion *I*_*j*_ is necessary to satisfy the following equation:

$$\begin{array}{c} R\left(f_{k}\right)≅\sum_{j=1}^{N}d_{j}\beta_{j}, \end{array}$$ (2)

where R is the Radon transform operator and $\beta_{j}=R\left(I_{j}\right)$.

The optimal coefficients *d*_*j*_ are estimated by solving the following minimization problem:

$$\begin{array}{c} d=\text{arg}\underset{d_{j}\geq 0}{\text{min}}∥p_{0}-\overset{N}{\underset{i=1}{\sum }}d_{j}\beta_{j}{∥}_{T_{M}^{c}}+\alpha ∥\nabla p_{0}-\overset{N}{\underset{i=1}{\sum }}d_{j}\nabla \beta_{j}{∥}_{\partial T_{M}}, \end{array}$$ (3)

where **d** = (*d*_1_, …, *d*_*N*_)^*T*^, $T_{M}^{c}$ is the complement of *T*_*M*_, and ∂*T*_*M*_ is the boundary of *T*_*M*_. The first term in Eq (3) minimizes the mismatch between the original sinogram and the linearly combined sinogram on $T_{M}^{c}$, and the second term regulates the smoothness along the boundary with the regularization parameter *α* > 0. In this work, the minimization problem is solved using Nelder-Mead simplex direct search [30] with *α* = 0.1. The mean value of each segmented subregion is used as an initial guess for solving Eq (3). With computed coefficients *d*_*j*_, a new linearly combined sinogram $\hat{p}=\sum_{j}\;d_{j}\beta_{j}$ is generated.

If the segmentation is performed without errors and the coefficients *d*_*j*_ are estimated correctly, $\hat{p}$ is equivalent to *p*_0_ on $T_{M}^{c}$ with ideal data acquisition condition. However, real projectin data are polychromatic and contain some amount of scatter, and thus residual errors (i.e., $\delta =p_{0}-\hat{p}$) still exist on $T_{M}^{c}$; thus, using data in the metal trace region of $\hat{p}$ is suboptimal for metal artifact reduction. In step three, the residual errors in the metal trace region are compensated by adding a linearly interpolated sinogram $\hat{\delta }$ of *δ* on the metal trace region and $\hat{p}$. The new sinogram $\hat{p}+\hat{\delta }$ coincides with the original sinogram on $T_{M}^{c}$ since $\hat{\delta }$ is equivalent to *δ* on $T_{M}^{c}$.

Then, *k* is updated by *k* + 1, and steps two and three are repeated using the reconstructed image *f*_*k*_ of $\hat{p}+\hat{\delta }$. This process continues until the norm of the residual errors ${∥\delta ∥}_{T_{M}^{c}}$ is less than tolerance *τ*.

In NMAR, interpolation errors in the metal trace region are minimized by using a normalized sinogram. If the object is piecewise constant and the X-ray spectrum is monochromatic, NMAR and the proposed method produce the same MAR results. When the object is heterogeneous and the X-ray spectrum is polychromatic, the denormalization step of NMAR may amplify the interpolation errors. This effect will be more significant when the metals exist on the surface of the object. In contrast, our approach is more stable since the interpolation errors are minimized by error subtraction. This effect will be described more clearly in our result section.

### Experiments

Both numerical and physical experiments were conducted to evaluate the performance of the proposed MAR algorithm.

#### Numerical simulations

**Fig 4:**
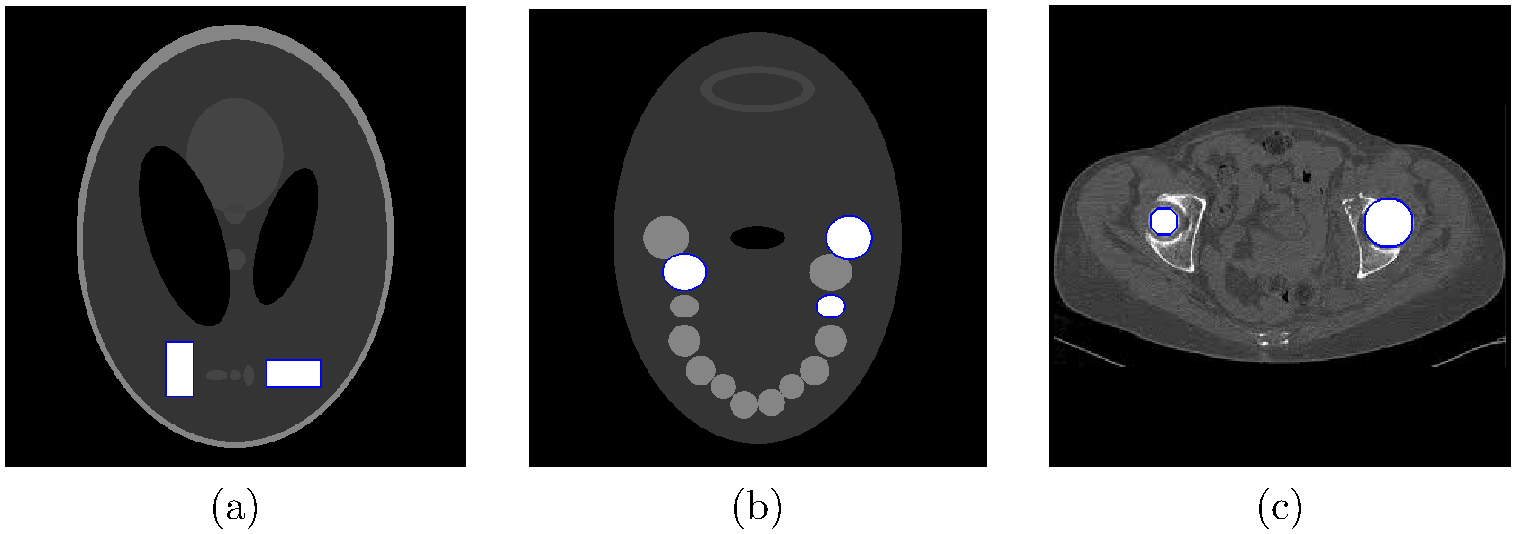
Three numerical phantoms. (a) Shepp-Logan phantom with two rectangular gold inserts indicated by blue squares, (b) jaw phantom with three gold implants indicated by blue circles, and (c) abdomen phantom with two gold implants indicated by blue circles.

The numerical simulations were performed with a fan beam CT geometry using Shepp-Logan, jaw and abdomen phantoms. A Shepp-Logan phantom (shown in Fig 4(a)) contains two rectangular gold inserts, a jaw phantom (shown in Fig 4(b)) contains three gold implants, and an abdomen phantom (Fig 4(c)) has two gold implants. For each phantom, polychromatic projection data were acquired with a 120 kVp tube voltage using the Siemens X-ray spectrum [31]. Attenuation coefficients of muscle, bone, and gold were obtained from the NIST X-ray Attenuation Database (ICRU-44) [32]. Each detector cell received 10^6^ photons without the object, and Poisson noise was simulated. Each phantom consists of 512 × 512 pixels with a 0.2 × 0.2 *mm*^2^ pixel size. To obtain the reference images, noiseless projection data of tissues and bones were reconstructed using a polychromatic X-ray spectrum. The simulation parameters are summarized in Table 1.

**Table 1:** Simulation parameters.

Parameter	Value
Source to iso-center distance	929.19, *mm*
Detector to iso-center distance	525.24, *mm*
Detector pixel size	0.388 × 0.388 *mm*^2^
Detector array size	1024
X-ray focal spot size	0.6 × 0.6 *mm*^2^
Number of source and detector lets	17 × 17
Rotation	circular 360°
Number of views	1080
Reconstructed image size	102.4 × 102.4 *mm*^2^
Reconstructed pixel size	0.2 × 0.2 *mm*^2^ (512 × 512)

#### Physical experiments

**Fig 5:**
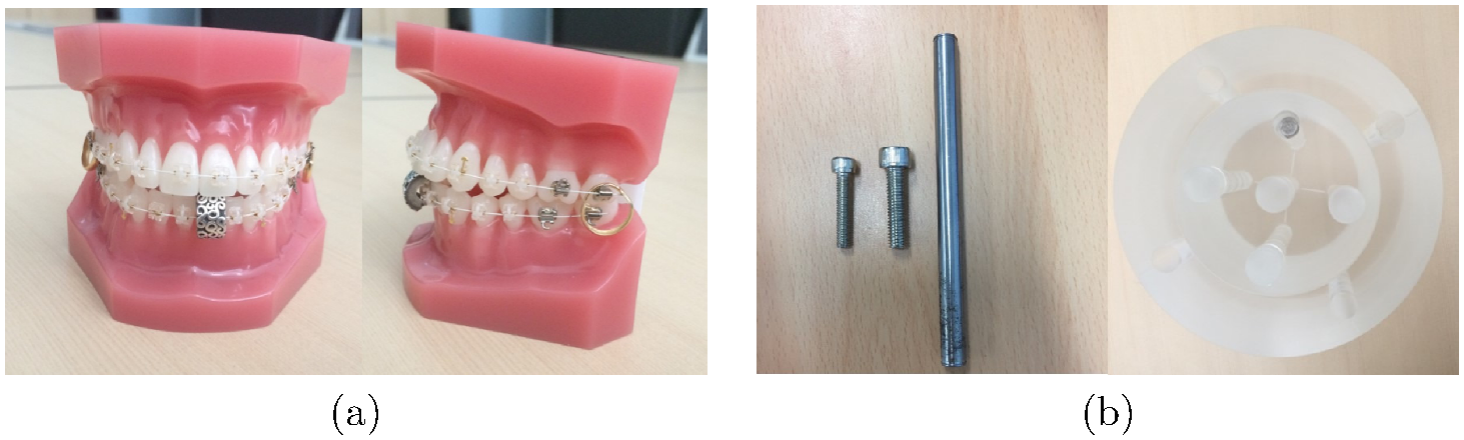
Two physical phantoms. (a) Tooth phantom containing an orthodontic bracket and three metals, and (b) PMMA phantom with a metal bar and two screws.

For the physical experiments, a tooth and polymethylmethacrylate (PMMA) phantoms were used. As shown in Fig 5(a), the tooth phantom consists of an orthodontic bracket with one silver clip at the fore tooth and two gold rings at the cheek tooth. A PMMA phantom consists of a 10 *cm*-diameter cylinder with five holes and a 16 *cm* diameter extension annulus with four holes (Fig 5(b)). A metal bar was placed at the center of the PMMA phantom, and two screws were placed at the edge of the PMMA phantom. Projection data were acquired from the bench-top cone beam CT (CBCT) system. The bench-top system includes a generator (Indico 100, CPI Communication & Medical Products Division, Georgetown, ON, Canada), a tungsten target X-ray source (Varian G-1592, Varian X-ray Product, Salt Lake City, UT, USA) with a 0.6 × 0.6 *mm*^2^ focal spot, and a 400 × 300 *mm*^2^ flat-panel detector (PaxScan 4030CB, Varian Medical Systems, Salt Lake City, UT, USA) with a tube voltage of 120 kVp and current of 5 mA. The experimental parameters are summarized in Table 2.

**Table 2:** Experiment parameters.

Parameter	Value
Tube Voltage	120 *kVp*
Current	5 *mA*
Source to iso-center distance	929.19 *mm*
Detector to iso-center distance	525.24 *mm*
Detector pixel size	0.388 × 0.388 *mm*^2^
Detector array size	1024 × 768
Rotation	circular 360°
Number of views	1080
Reconstructed volume size	204.8 × 204.8 × 51.2 *mm*^3^
Reconstructed voxel size	0.2 × 0.2 × 0.2 *mm*^3^ (1024 × 1024 × 256)

### Performance comparison

We compared the performance of the proposed algorithm with LIN [6] and NMAR [18]. The performance of NMAR was evaluated using three different prior images: prior image by simple thresholding (NMAR_*th*_), prior image by RAC segmentation of LIN (NMAR_*seg*_*s*__), and the same prior image with the proposed algorithm (NMAR_*seg*_*e*__). In NMAR_*th*_, the thresholding value was chosen by the attenuation coefficient of the material (i.e., air = 0, soft tissue = 0.28, and bone = 0.4). In NMAR_*seg*_*s*__ and NMAR_*seg*_*e*__, the coefficients of the prior images were chosen as the mean value of the corresponding subregions.

For the quantitative evaluation, the mean square error (MSE) and standard deviation were used. For the simulation data, the MSE between the reference image and each MAR image was computed using the entire image except the metal region. For the experimental phantoms, the region of interest (ROI) was selected within homogeneous regions, and the standard deviation was calculated for each MAR image.

## Results

### Numerical simulations

**Fig 6:**
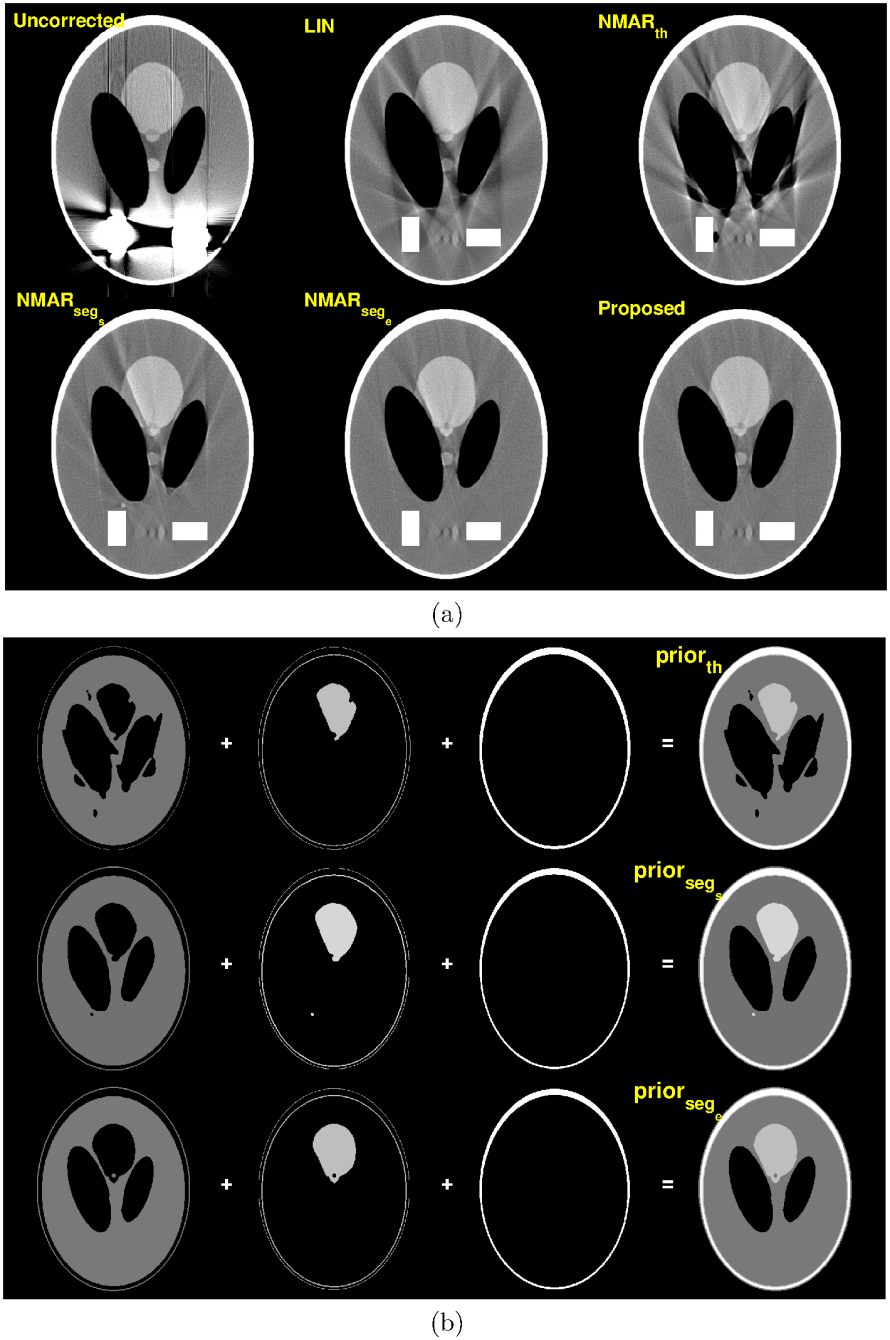
Results of the Shepp-Logan phantom (C = 250 HU/W = 750 HU). (a) Top row: reconstruction images by (left) Uncorrected, (middle) LIN, and (right) NMAR_*th*_ images. Bottom row: reconstruction images by (left) NMAR_*seg*_*s*__, (middle) NMAR_*seg*_*e*__, and (right) the proposed MAR. (b) Prior images by (top) simple thresholding, (middle) RAC segmentation of the LIN image, and (bottom) RAC segmentation of the final iteration image.

Fig 6(a) shows the MAR results of the Shepp-Logan phantom. Due to significant beam hardening artifacts in the uncorrected image, high quality prior images could not be acquired by simple thresholding (shown in Fig 6(b)). As a result, the NMAR_*th*_ image contained severe streak artifacts. Using good prior images improved the image quality as shown in the NMAR_*seg*_*s*__ image, and further improvement was observed in the NMAR_*seg*_*e*__ image. It is also shown that the proposed MAR image shows similar performance to the NMAR_*seg*_*e*__ image since the error amplification in the denormalization step of NMAR is not significant because of the negligible beam hardening effect of the soft tissues in the original sinogram.

**Fig 7:**
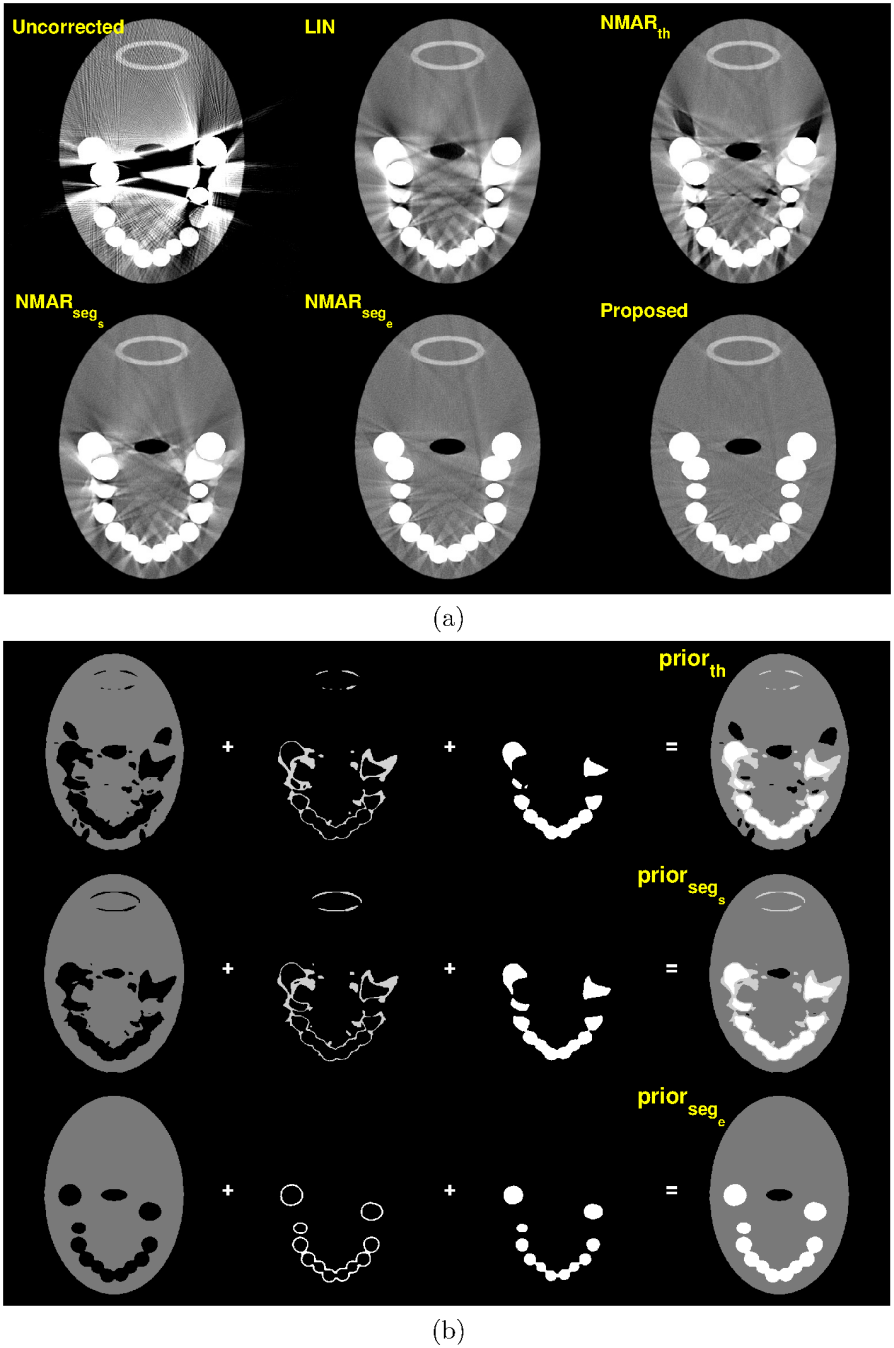
Results of the jaw phantom (C = 250 HU/W = 750 HU). (a) Top row: reconstruction images by (left) Uncorrected, (middle) LIN, and (right) NMAR_*th*_ images. Bottom row: reconstruction images by (left) NMAR_*seg*_*s*__, (middle) NMAR_*seg*_*e*__, and (right) the proposed MAR. (b) Prior images by (top) simple thresholding, (middle) RAC segmentation of the LIN image, and (bottom) RAC segmentation of the final iteration image.

**Fig 8:**
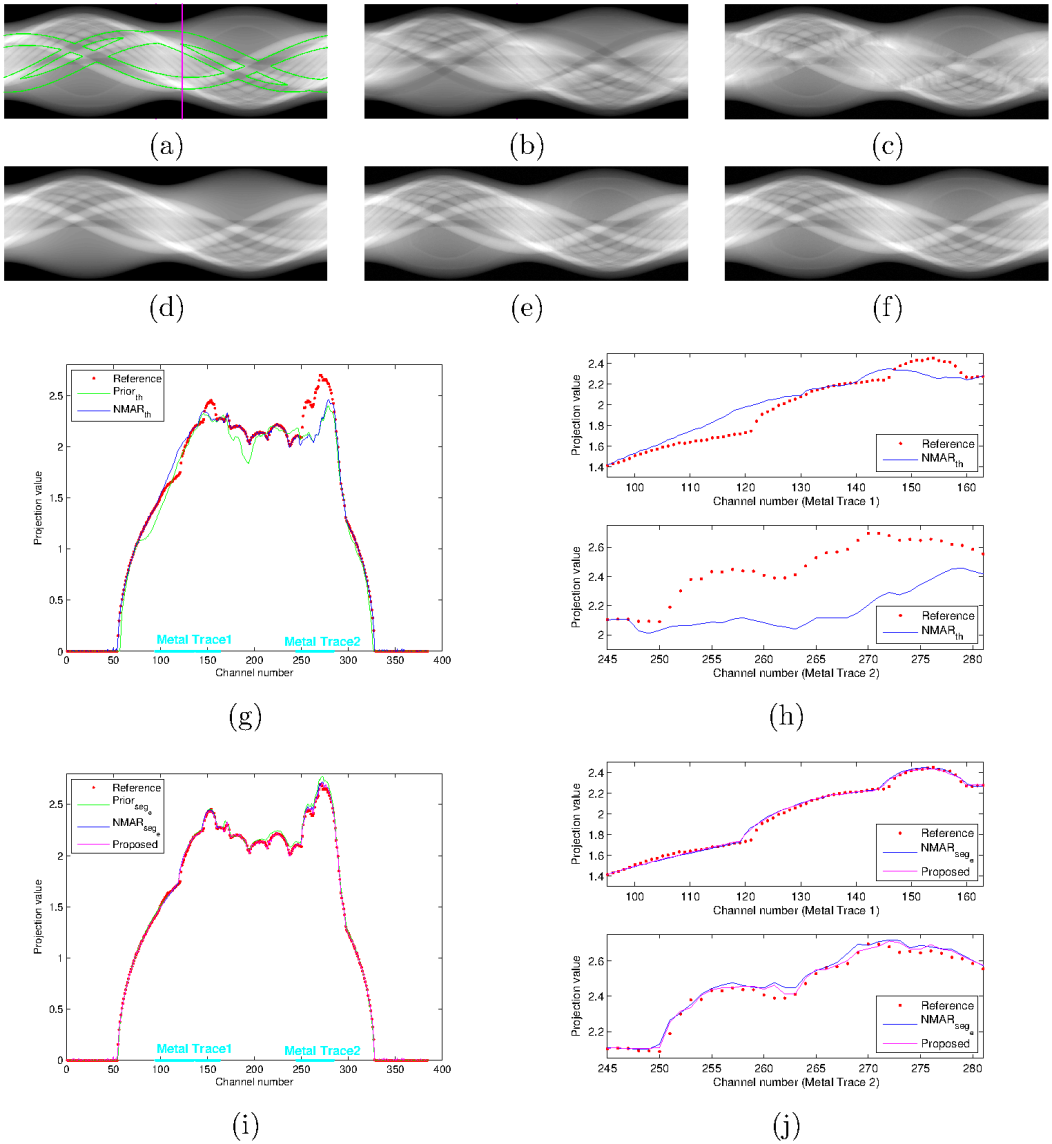
Sinogram profiles. (a) reference, (b) Prior_*th*_, (c) NMAR_*th*_, (d) Prior_*seg*_*e*__, (e) NMAR_*seg*_*e*__, and (f) the proposed algorithm. Comparison of sinogram profiles of (g) reference, Prior_*th*_, and NMAR_*th*_, and (h) enlarged version of the profiles within metal traces 1 and 2 of Fig 8(g). Comparison of sinogram profiles of (i) reference, Prior_*seg*_*e*__, NMAR_*seg*_*e*__, and the proposed algorithm, and (j) enlarged version of the profiles within metal traces 1 and 2 of Fig 8(i).

Fig 7(a) shows the MAR results of the jaw phantom and Fig 7(b) compares the prior images acquired by a simple thresholding and RAC technique. Unlike the Shepp-Logan results, the proposed MAR method shows better performance than the NMAR_*seg*_*e*__ image. Fig 8(a)–8(f) show the sinogram of the reference image, prior images by simple thresholding, the NMAR_*th*_ image, prior images by the RAC technique, the NMAR_*seg*_*e*__ image, and the proposed MAR image, respectively. Profiles of the sinogram images (indicated by the magenta line in Fig 8(a)) are also compared. As shown in Fig 8(g) and 8(h), the NMAR_*th*_ produces significant errors within the metal trace region. Using good prior images reduces the estimation errors in the metal trace region as shown in Fig 8(i). However, residual errors still exist in the profile of NMAR_*seg*_*e*__ as shown in Fig 8(j). Note that the original sinogram contains beam hardening effects generated from multiple bones; thus, the denormalization step of NMAR amplifies the interpolation errors on the metal trace region. The proposed MAR algorithm reduces the residual errors by the additional residual error correction step (i.e., Step 3 in Fig 3), and thus, further improvement in MAR can be achieved.

**Fig 9:**
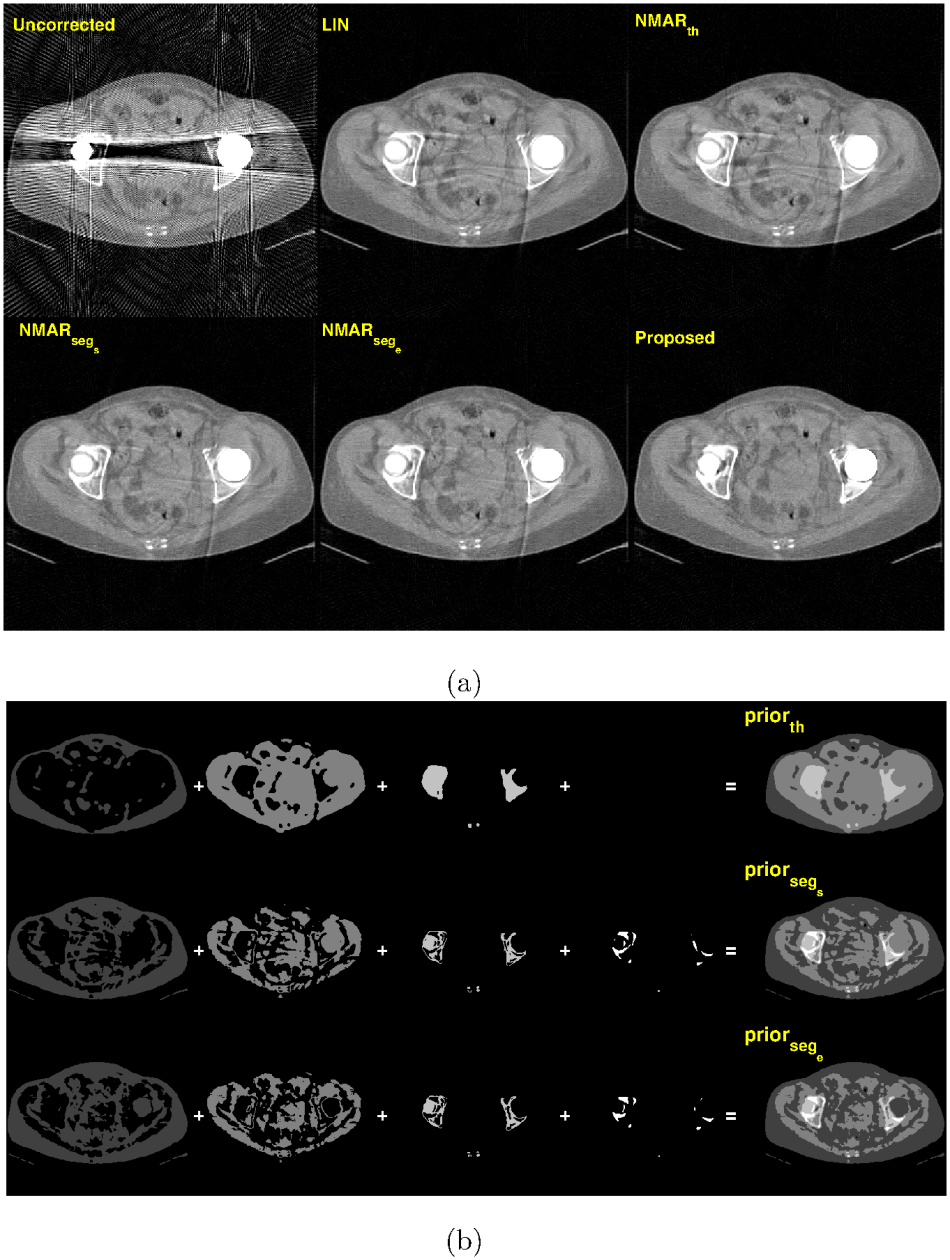
Results of the abdomen phantom (C = 500 HU/W = 1500 HU). (a) Top row: reconstruction images by (left) Uncorrected, (middle) LIN, and (right) NMAR*_th_* images. Bottom row: reconstruction images by (left) NMAR_*seg*_*s*__, (middle) NMAR_*seg*_*e*__, and (right) the proposed MAR. (b) Prior images by (top) simple thresholding, (middle) RAC segmentation of the LIN image, and (bottom) RAC segmentation of the final iteration image.

Fig 9(a) shows the MAR results of the abdomen phantom. Compared to the previous numerical phantoms, the abdomen phantom is more heterogeneous, and thus more accurate prior images would be required to reduce metal artifacts effectively. However, due to the severe noise and metal artifacts in the uncorrected image, prior images generated by simple thresholding miss details of inner structures in the abdomen phantom (shown in Fig 9(b)). As a result, the NMAR_*th*_ image contains severe streak artifacts. In contrast, prior images generated by the RAC technique contain more details, which improve the performance of the NMAR. While all other MAR algorithms contain residual streaks artifacts between two metals, the proposed algorithm reduces them effectively.

In simulations, the number of prior images was chosen by RAC automatically, and the tolerance *τ* was set to 10% of the first norm of the residual errors. Table 3 summarizes the number of prior image and coefficients for numerical simulations, and Table 4 summarizes the MSE values for different MAR images. For all numerical phantoms, the proposed algorithm shows the best performance in metal artifact reduction.

**Table 3:** Number of prior image *N* and coefficients *d*_*j*_ in numerical simulations.

	Method	*N*	*d*_1_	*d*_2_	*d*_3_	*d*_4_
Shepp-Logan	NMAR_*th*_	3	0.230	0.311	0.603	
	NMAR_*seg*_*s*__	3	0.226	0.340	0.614	
	NMAR_*seg*_*e*__	3	0.226	0.333	0.612	
	Proposed	3	0.234	0.309	0.655	
Jaw	NMAR_*th*_	3	0.269	0.331	0.525	
	NMAR_*seg*_*s*__	3	0.233	0.327	0.512	
	NMAR_*seg*_*e*__	3	0.241	0.441	0.530	
	Proposed	3	0.236	0.538	0.630	
Abdomen	NMAR_*th*_	3	0.248	0.321	0.485	
	NMAR_*seg*_*s*__	4	0.270	0.337	0.426	0.513
	NMAR_*seg*_*e*__	4	0.259	0.328	0.474	0.621
	Proposed	4	0.294	0.575	0.685	0.757

**Table 4:** MSE of numerical simulations.

	LIN	NMAR_*th*_	NMAR_*seg*_*s*__	NMAR_*seg*_*e*__	Proposed
Shepp Logan	127.9	195.9	85.9	62.4	59.4
Jaw	280.2	245.1	242.9	110.4	77.5
Abdomen	64.4	66.1	57.8	56.0	54.7

### Physical experiment

**Fig 10:**
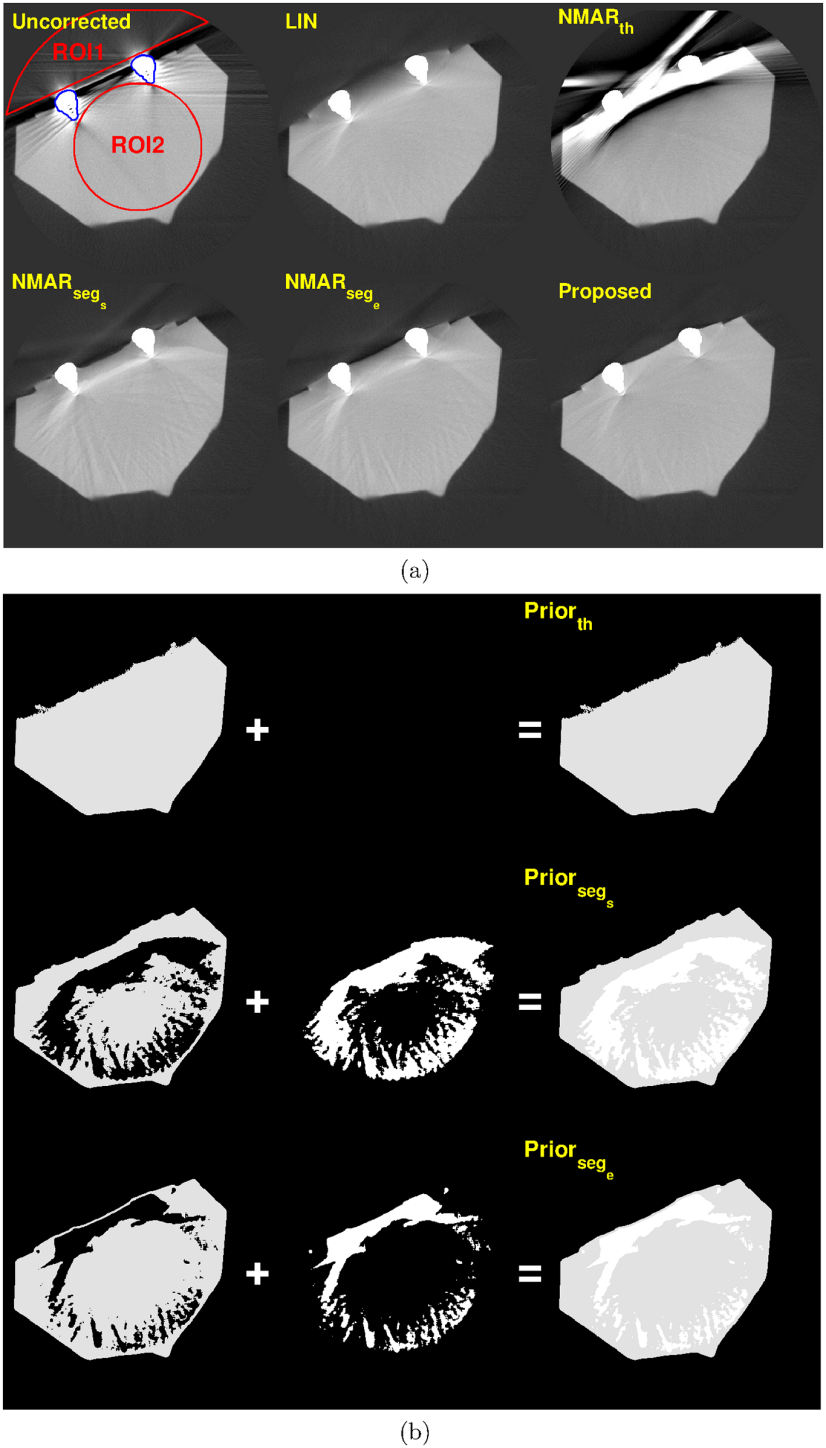
Results of tooth phantom with orthodontic bracket, on slice # 17 (C = -250 HU/W = 1250 HU). (a) Top row: reconstruction images by (left) Uncorrected, (middle) LIN, and (right) NMAR_*th*_ images. Bottom row: reconstruction images by (left) NMAR_*seg*_*s*__, (middle) NMAR_*seg*_*e*__, and (right) the proposed results. ROI 1 and ROI 2 are indicated by red lines on the uncorrected image. (b) Prior images by (top) simple thresholding, (middle) RAC segmentation of LIN image, and (bottom) RAC segmentation of final iteration image.

For the tooth phantom, three slices from the reconstructed three-dimensional volume were chosen. Fig 10 shows MAR results and prior images of slice 17. Notice that the NMAR_*th*_ image contains significant streak artifacts owing to the poor quality of the prior images. Although the streak artifacts are reduced in the NMAR_*seg*_*s*__ and NMAR_*seg*_*e*__ images, residual streak artifacts are still observed. Since the tooth phantom contains the metals on the surface of the object, small interpolation errors in the metal trace region are significantly amplified during the denormalization step of the NMAR algorithm. In contrast, the proposed MAR algorithm effectively reduces the streak artifacts.

**Fig 11:**
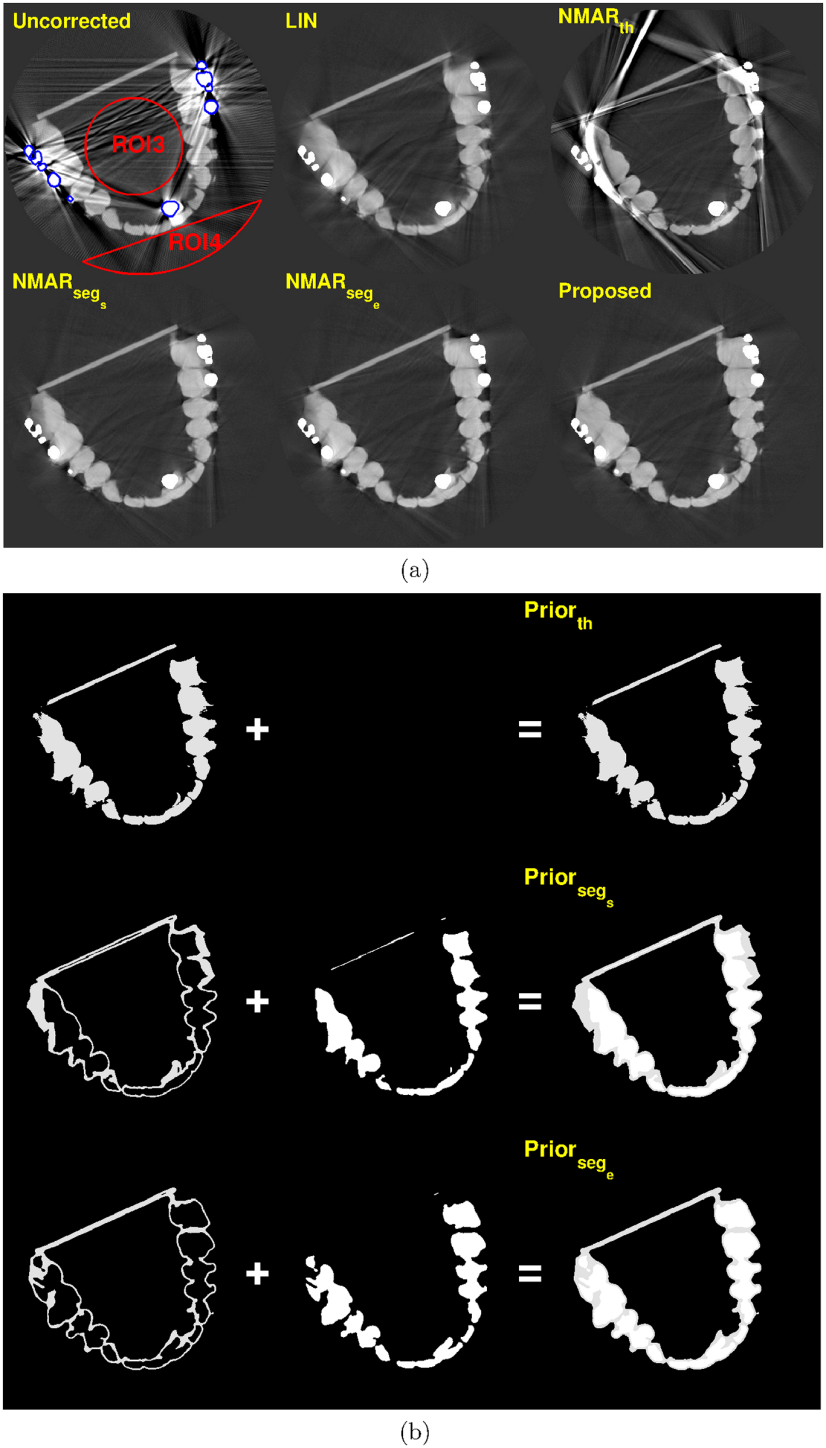
Results of tooth phantom with orthodontic bracket, on slice # 114 (C = -250 HU/W = 1250 HU). (a) Top row: reconstruction images by (left) Uncorrected, (middle) LIN, and (right) NMAR_*th*_ images. Bottom row: reconstruction images by (left) NMAR_*seg*_*s*__, (middle) NMAR_*seg*_*e*__, and (right) the proposed results. ROI 3 and ROI 4 are indicated by red lines on the uncorrected image. (b) Prior images by (top) simple thresholding, (middle) RAC segmentation of LIN image, and (bottom) RAC segmentation of final iteration image.

**Fig 12:**
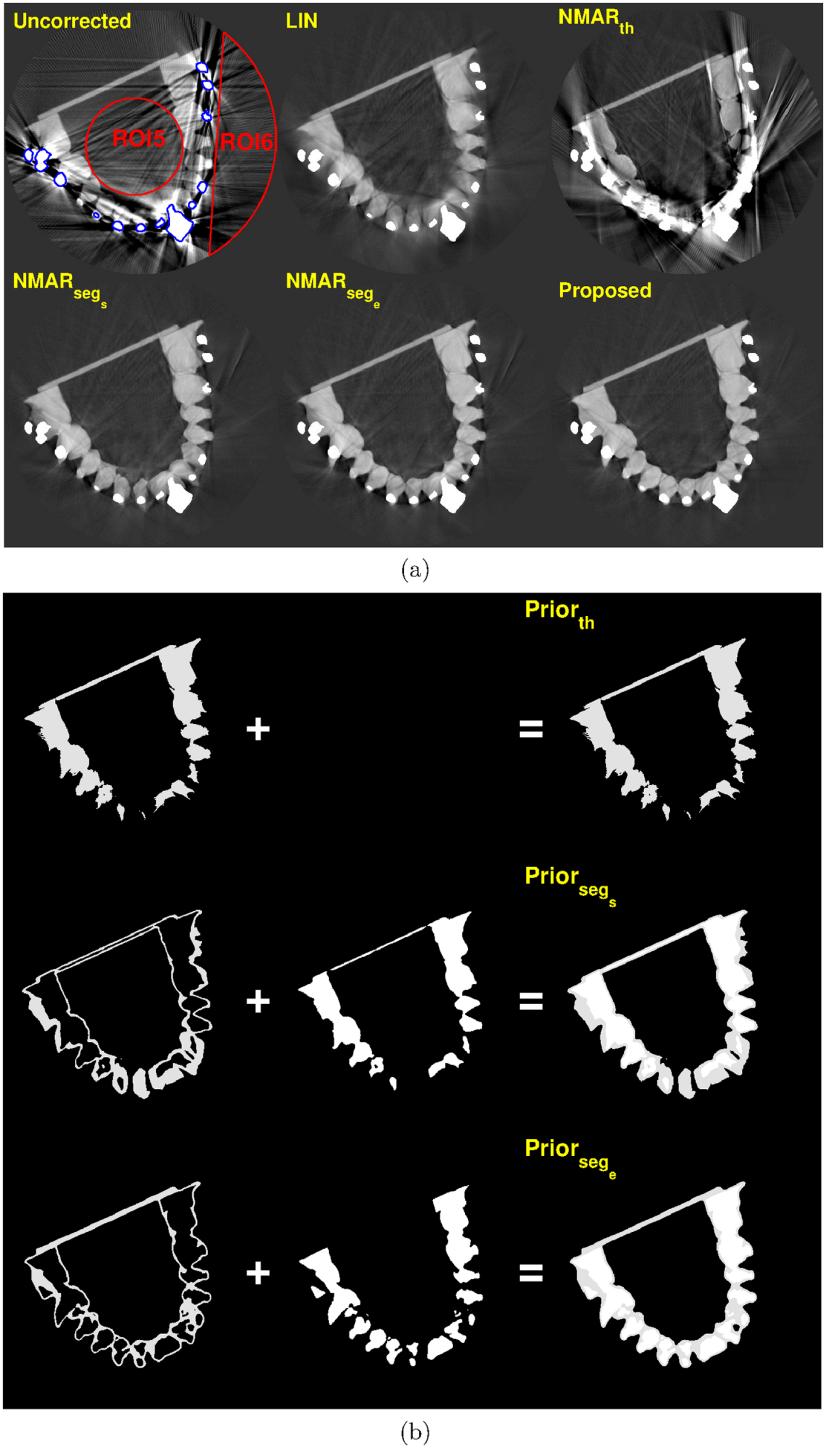
Results of tooth phantom with orthodontic bracket, on slice # 155 (C = -250 HU/W = 1250 HU). (a) Top row: reconstruction images by (left) Uncorrected, (middle) LIN, and (right) NMAR_*th*_ images. Bottom row: reconstruction images by (left) NMAR_*seg*_*s*__, (middle) NMAR_*seg*_*e*__, and (right) the proposed results. ROI 5 and ROI 6 are indicated by red lines on the uncorrected image. (b) Prior images by (top) simple thresholding, (middle) RAC segmentation of LIN image, and (bottom) RAC segmentation of final iteration image.

Figs 11 and 12 show the MAR results and prior images of slice 114 and 155, respectively, where multiple metals are present. Compared to the other MAR algorithms, the proposed method reduces metal artifacts more effectively.

For the quantitative evaluation, two ROIs were chosen on the homogeneous region, which is indicated by the red lines in Figs 10(a)–12(a). The standard deviation of each ROI was calculated and summarized in Table 5. For the tooth phantom, the proposed MAR algorithm has the smallest standard deviation for all three slices.

**Table 5:** Standard deviation of the ROIs for physical phantoms.

	slice #		Ori	LIN	NMAR_*th*_	NMAR_*seg*_*s*__	NMAR_*seg*_*e*__	Proposed
Tooth	#17	ROI1	195.7	33.1	769.9	54.4	43.4	25.8
		ROI2	105.0	59.7	651.3	87.7	64.6	41.2
	#114	ROI3	252.9	59.4	40.1	40.0	40.5	40.6
		ROI4	151.0	46.0	206.1	40.8	44.2	32.4
	#155	ROI5	233.1	94.6	153.5	82.0	84.2	81.4
		ROI6	232.2	64.3	306.9	50.8	42.5	38.4
PMMA		ROI1	313.8	78.3	76.2	73.4	75.9	75.7
		ROI2	352.4	75.6	69.4	69.6	70.7	69.3
		ROI3	127.9	74.0	66.4	65.8	66.3	66.6
		ROI4	129.7	71.3	69.0	69.3	71.1	68.7

**Fig 13:**
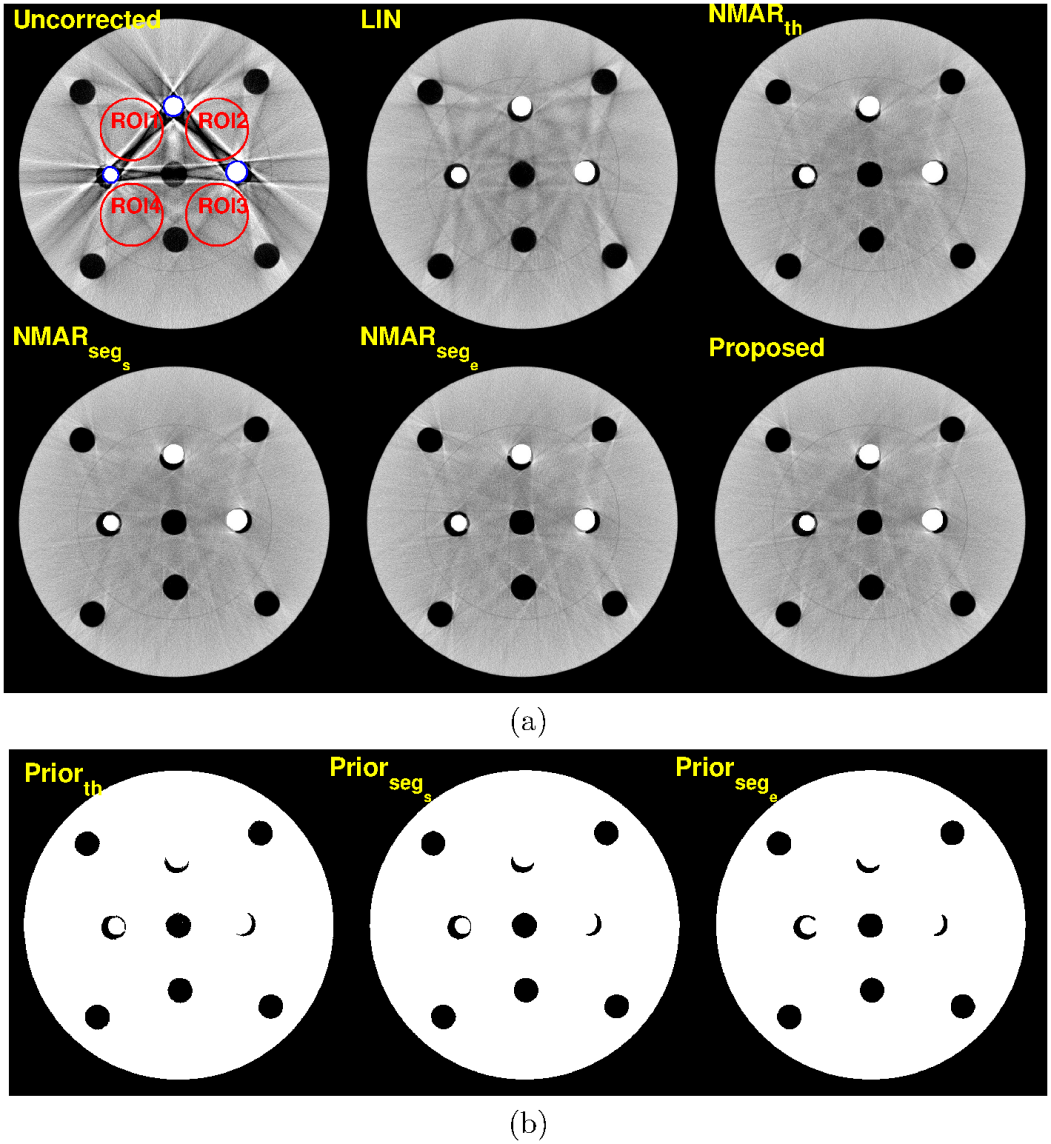
Results of a PMMA phantom (C = 0 HU/W = 600 HU). (a) Top row: reconstruction images by (left) Uncorrected, (middle) LIN, and (right) NMAR_*th*_ images. Bottom row: reconstruction images by (left) NMAR_*seg*_*s*__, (middle) NMAR_*seg*_*e*__, and (right) the proposed results. ROI 1- ROI 4 are indicated by red lines on the uncorrected image. (b) Prior images. Left: prior by simple thresholding, middle: prior by RAC segmentation of the LIN image, right: prior by RAC segmentation of the final iteration image.

Fig 13 shows MAR results and prior images of the PMMA phantom with three metal inserts, where the central slice from the reconstructed three-dimensional volume is selected. Notice that the prior images by simple thresholding and the RAC technique are very similar and thus, all NMAR algorithms show similar MAR results with the proposed MAR algorithm.

For the quantitative evaluation, four ROIs were selected on the homogeneous region, which are indicated by red lines in Fig 13(a). The standard deviation of each ROI was calculated and summarized in Table 5. Notice that the three NMAR images and the proposed MAR image show similar standard deviation owing to the simple structure of the PMMA phantom. Table 6 summarizes the number of iterations for each phantom. More iterations are required when the image quality of LIN is poor. Table 7 summarizes the number of prior image and coefficients for each experimental phantom.

**Table 6:** Iteration numbers of the proposed algorithm.

	Shepp-Logan	Jaw	Abdomen	Tooth	PMMA
Iteration No.	19	37	3	5	2

**Table 7:** Number of prior image *N* and coefficients *d*_*j*_ in physical phantoms.

	Method	*N*	*d*_1_	*d*_2_
Tooth	NMAR_*th*_	1	0.208	
	NMAR_*seg*_*s*__	2	0.176	0.214
	NMAR_*seg*_*e*__	2	0.184	0.210
	Proposed	2	0.215	0.222
PMMA	NMAR_*th*_	1	0.198	
	NMAR_*seg*_*s*__	1	0.198	
	NMAR_*seg*_*e*__	1	0.198	
	Proposed	1	0.200	

## Discussion and conclusion

We presented a new MAR algorithm using multiple prior images with subsequential residual error correction. Traditional interpolation-based MAR algorithms replace the metal trace region with neighboring data depending on the distance, which introduces streak artifacts owing to interpolation errors in the metal trace region. The proposed method minimizes interpolation errors by utilizing multiple prior images generated by the RAC technique and additional residual error correction method in sinogram space. The performance of the proposed method was compared with LIN and NMAR with different prior images. For the homogeneous object, NMAR and the proposed method show similar performance in metal artifact reduction. However, when the object is complex with multiple bone objects, the proposed method shows better MAR results. For a complex object, we also showed that the performance of the NMAR algorithm can be improved significantly using prior images generated by the RAC technique.

In NMAR, interpolation errors in the metal trace region were reduced by performing the interpolation on the normalized sinogram. However, using poor quality prior images can degrade the performance of NMAR since the interpolation errors are magnified by the denormalization step of the NMAR algorithm. This effect is more significant when the metals lie on the surface of the objects. Using good prior images improves the performance of the NMAR algorithm significantly. As shown in the results of NMAR_*seg*_*s*__, RAC segmentation improves NMAR compared to NMAR_*th*_, but the residual errors caused by the denormalization step still exist, especially for complex objects containing multiple bone objects. In contrast, the proposed algorithm does not amplify interpolation errors since the metal trace region is filled in by the summation of linearly combined sinograms of prior images and subsequential residual error correction.

The proposed method iteratively reduces interpolation errors. Each iteration consists of prior image generation, sinogram basis generation, and residual error correction. Each iteration takes approximately 15 *s* for a two-dimensional image in MATLAB (MathWorks, Natick, MA) on a work station with an Intel XEON E5-2630V2 processor at 2.6GHz. The number of iterations depends on the object’s complexity and the quality of the LIN image. Since the proposed algorithm can be applied independently for each slice, the computation time for three-dimensional volume data would be similar to the two-dimensional case by parallel computing using a graphics processing unit (GPU). The computation time can be more accelerated by C/C++ implementation, which is essential for real clinical applications.

In this work, we treated the projection data in the metal trace region as missing or completely unreliable. However, when the density of metals is not high enough, the metal trace region may contain available information of the object, and thus, further improvement of the MAR can be achieved by frequency split MAR (FSMAR) [19]. Comparison of the image quality improvement with FSMAR with NMAR images and proposed MAR images is a subject of future research. In conclusion, we presented a new MAR algorithm using multiple prior images with additional residual error correction. The results show that the proposed algorithm outperforms other MAR algorithms, especially when the object is complex with multiple bone objects.

## Supporting information

S1 Fig 2D Shepp Logan phantom. Data related to Fig 6.
(MAT)

S2 Fig 2D jaw phantom. Data related to Fig 7.
(MAT)

S3 Fig 2D jaw phantom. Data related to Fig 8.
(MAT)

S4 Fig 2D abdomen phantom. Data related to Fig 9.
(MAT)

S5 Fig 3D orthodontic phantom. Data related to Figs 10–12.
(MAT)

S6 Fig 3D PMMA phantom. Data related to Fig 13.
(MAT)
